# Supplementary figures and images for: Conserved signalling functions for Mps1, Mad1 and Mad2 in the Cryptococcus neoformans spindle checkpoint
Source: PLoS Genet. 2024 Jun 3;20(6):e1011302. doi: 10.1371/journal.pgen.1011302 (PMC11175454; doi:10.1371/journal.pgen.1011302)

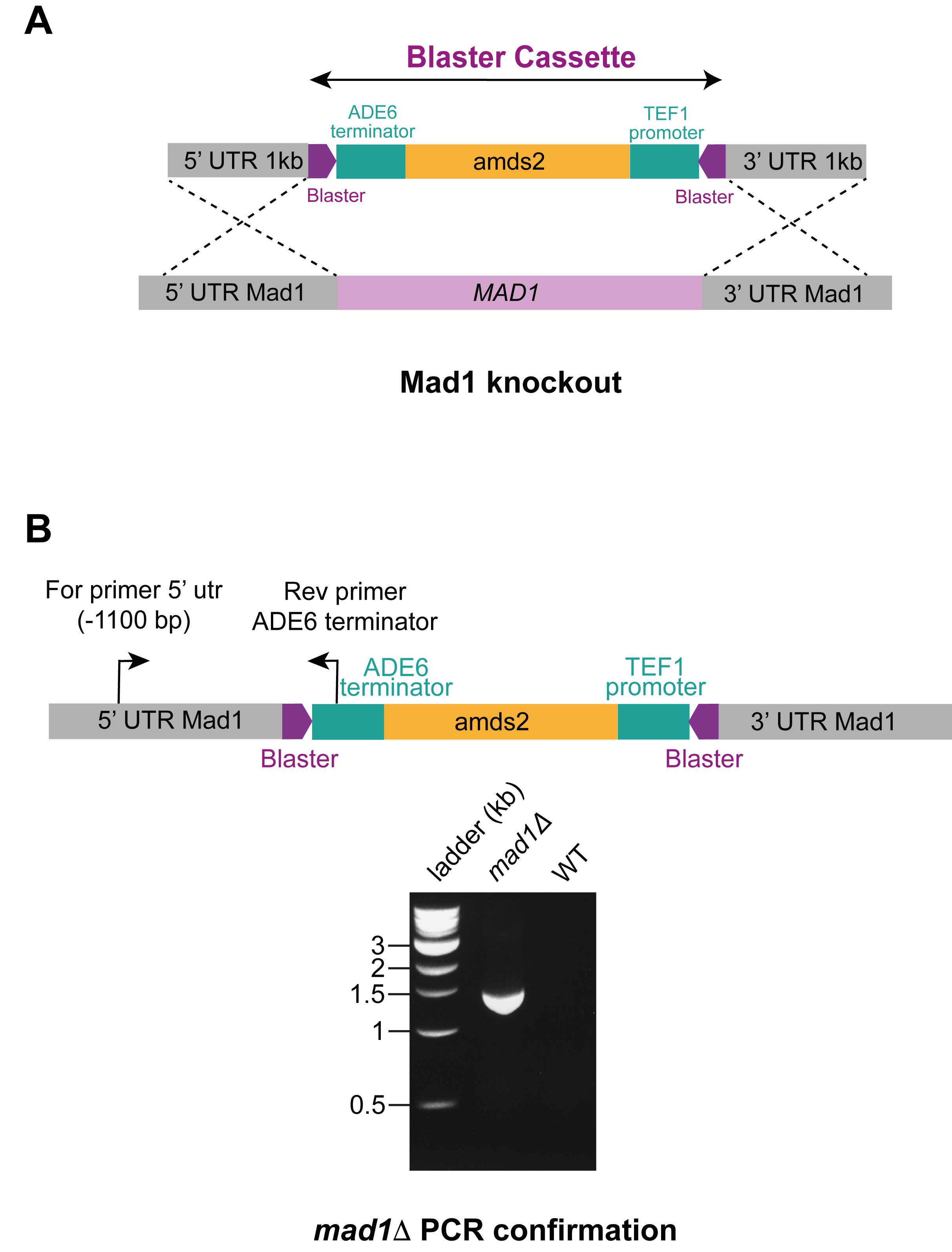

Supplement: S1 Fig — (A) Targeting schematic. (B) PCR analysis of genomic DNA confirms that the Blaster cassette had integrated at the CnMAD1 locus. (TIF) [file pgen.1011302.s001.tif]

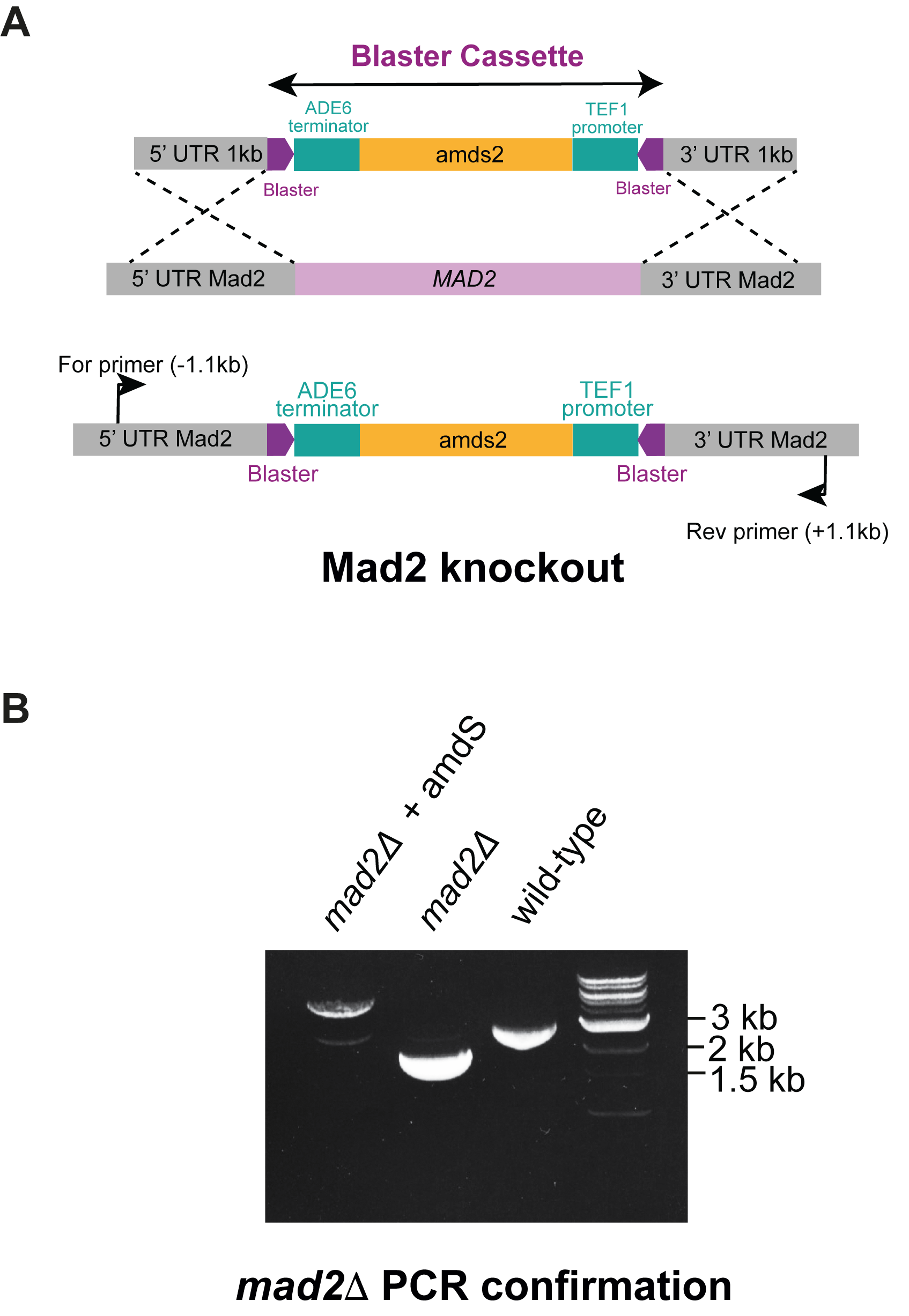

Supplement: S2 Fig — (TIF) [file pgen.1011302.s002.tif]

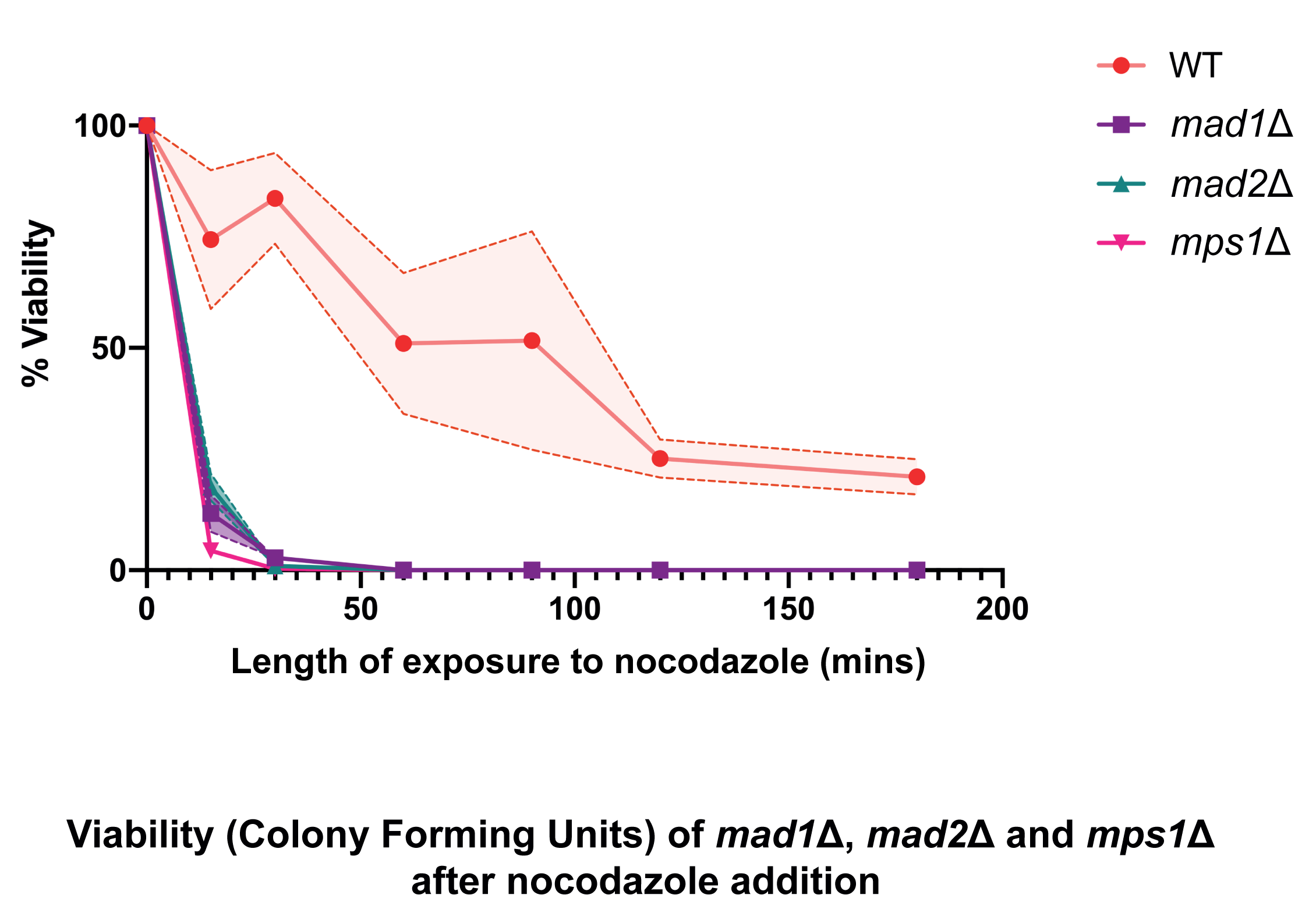

Supplement: S3 Fig — The four strains were grown to log phase and then nocodazole added to a final concentration of 2μg/ml. Cultures were then washed, diluted and plated onto YPD. Viable colonies were counted after 3 days growth. (TIF) [file pgen.1011302.s003.tif]

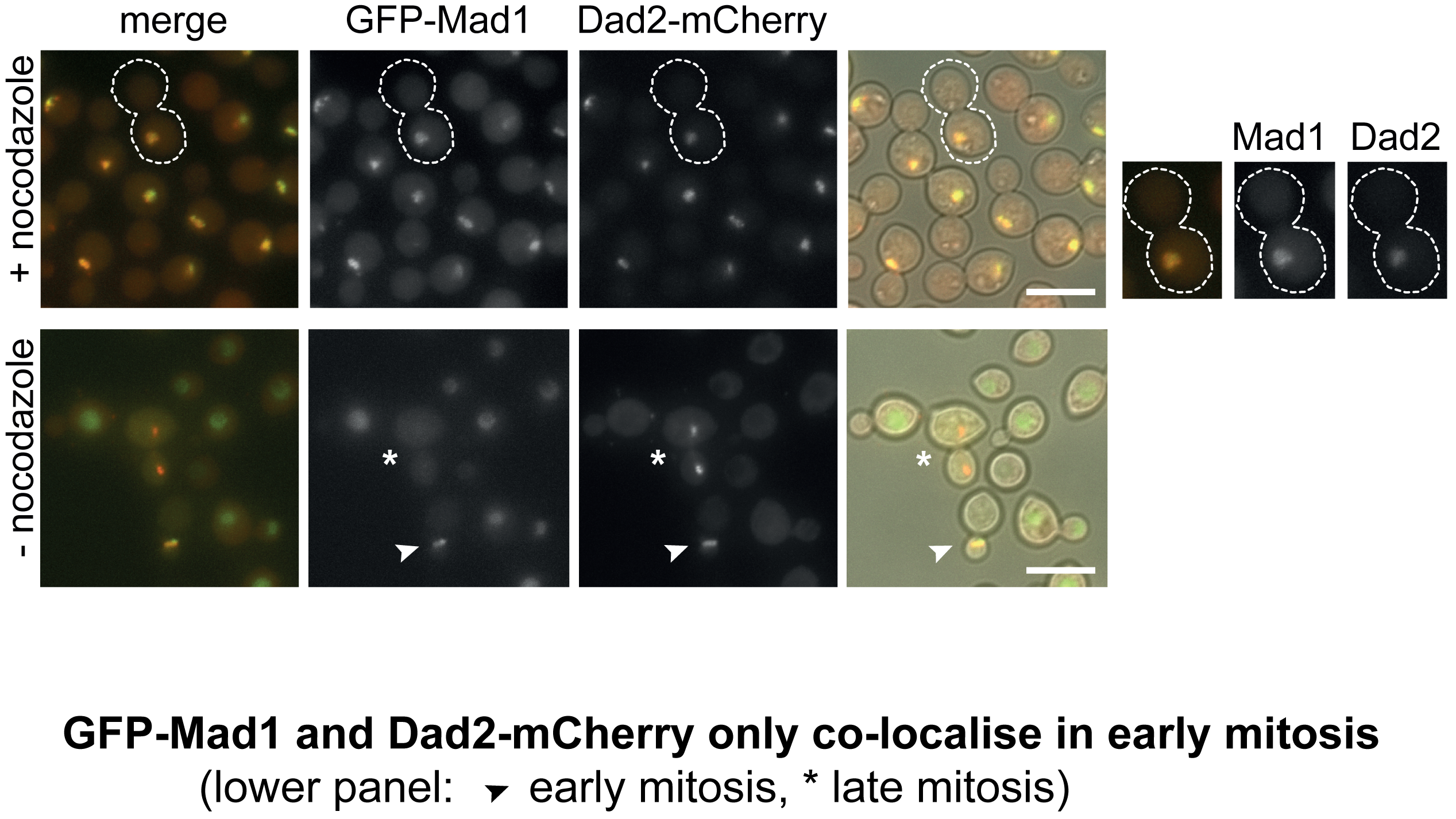

Supplement: S4 Fig — In nocodazole arrested cells, GFP-Mad1 does co-localise with the kinetochore marker mCherry-Dad2. In cycling cells they only co-localise early in mitosis (cell marked with arrow head), not in late mitosis (cell marked with *). Scale bar is 10μm. (TIF) [file pgen.1011302.s004.tif]

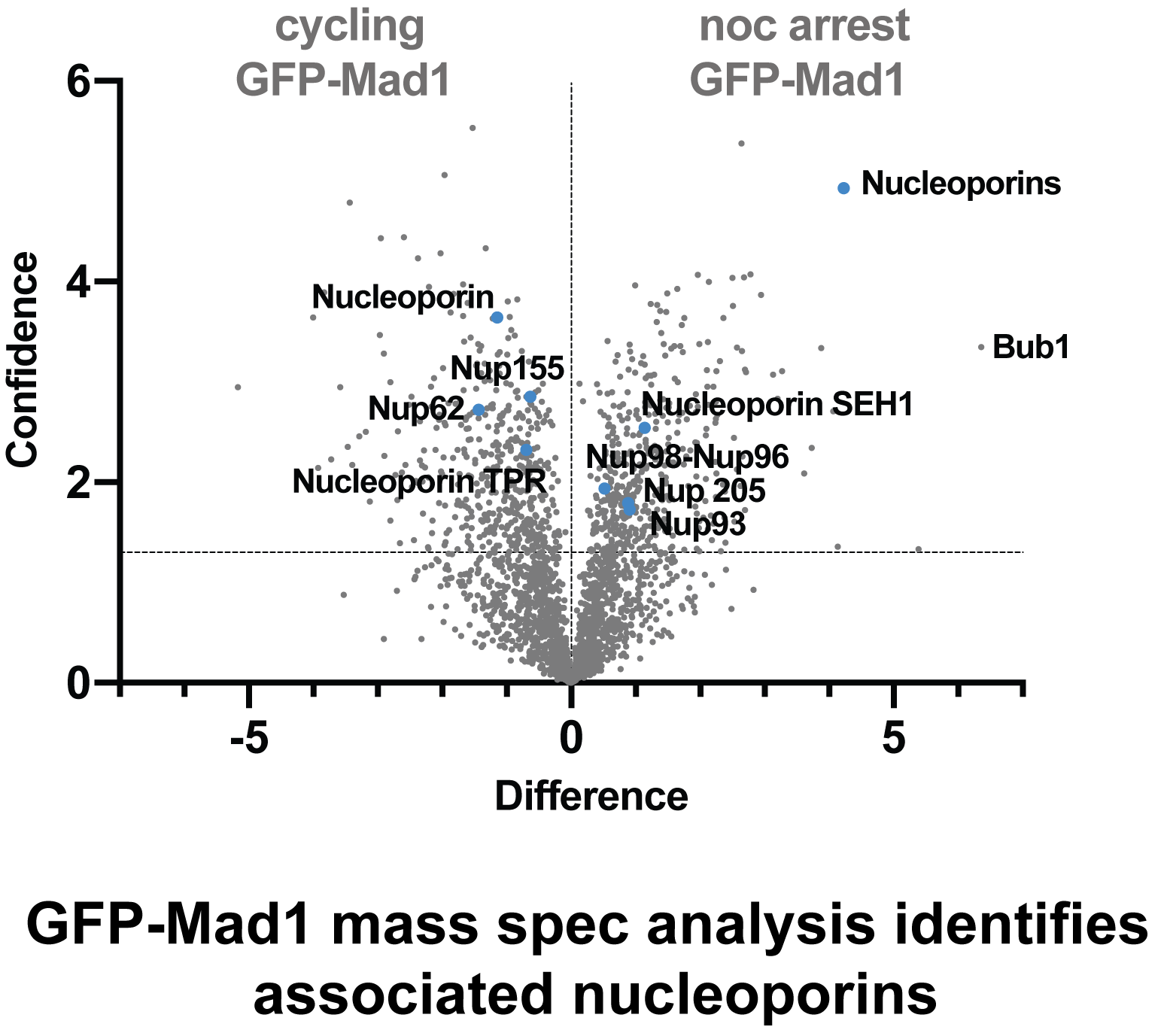

Supplement: S5 Fig — Volcano plots show the difference (mean LFQ difference) and confidence (-log10P-value of Perseus statistical test) between the cycling and nocodazole-arrested GFP-Mad1 pull-downs (n = 3 for each). This is the same dataset as in Fig 5B. Here nucleoporins are highlighted, some of which including TPR are enriched with the Mad1 pulldown in non-mitotic cells. (TIF) [file pgen.1011302.s005.tif]

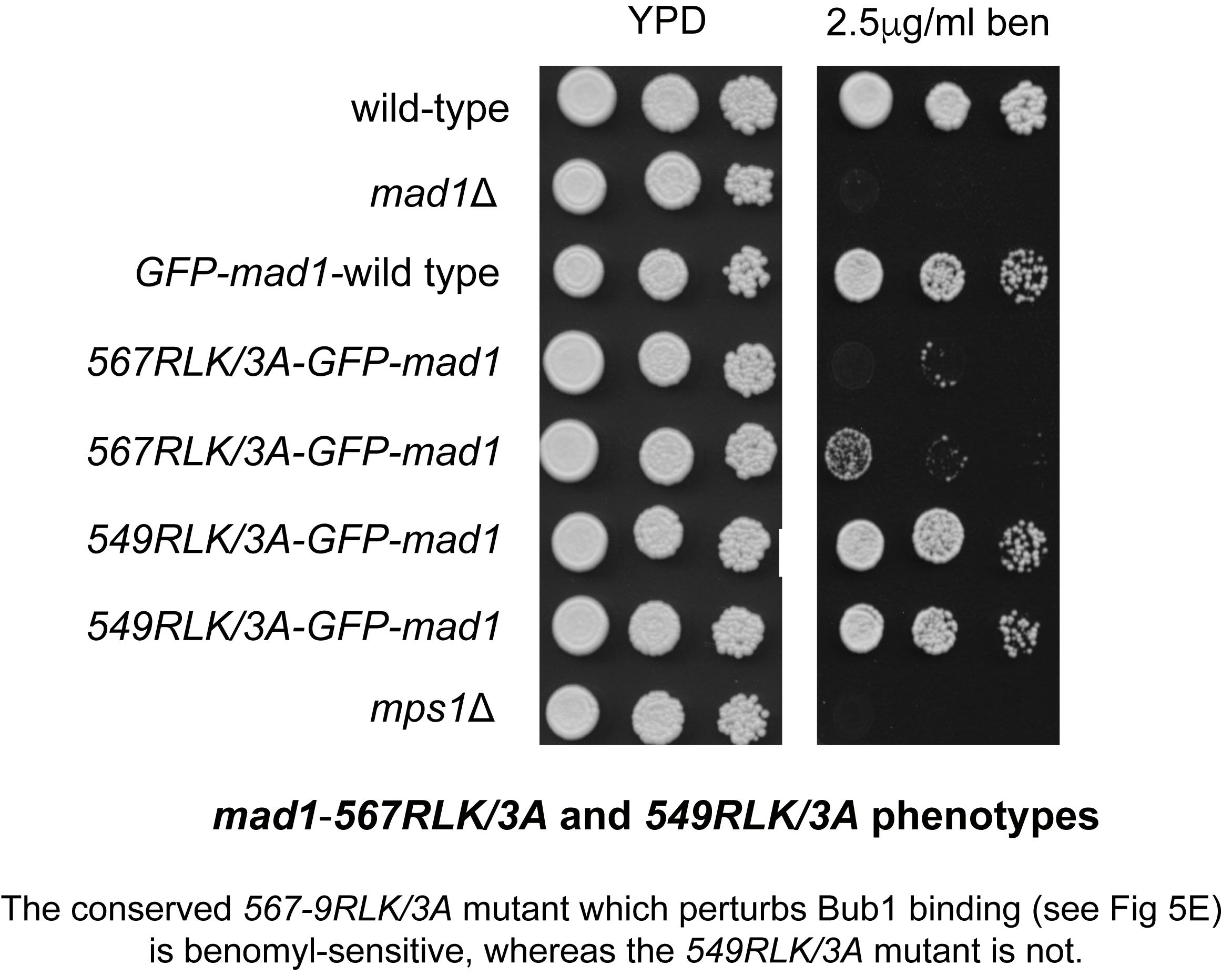

Supplement: S6 Fig — The conserved RLK motif in Mad1 (residues 567–9) is required for Bub1 binding (Fig 5E) and when mutated generates a benomyl sensitive strain. Mad1 RLK residues 549–551 are not conserved, are not required for Bub1 binding (Fig 5E) and do not produce a benomyl-sensitive strain when mutated. The strains indicated were serially diluted and plated onto YPD plates with and without benomyl. Images were taken after 3 days growth at 30. (TIF) [file pgen.1011302.s006.tif]

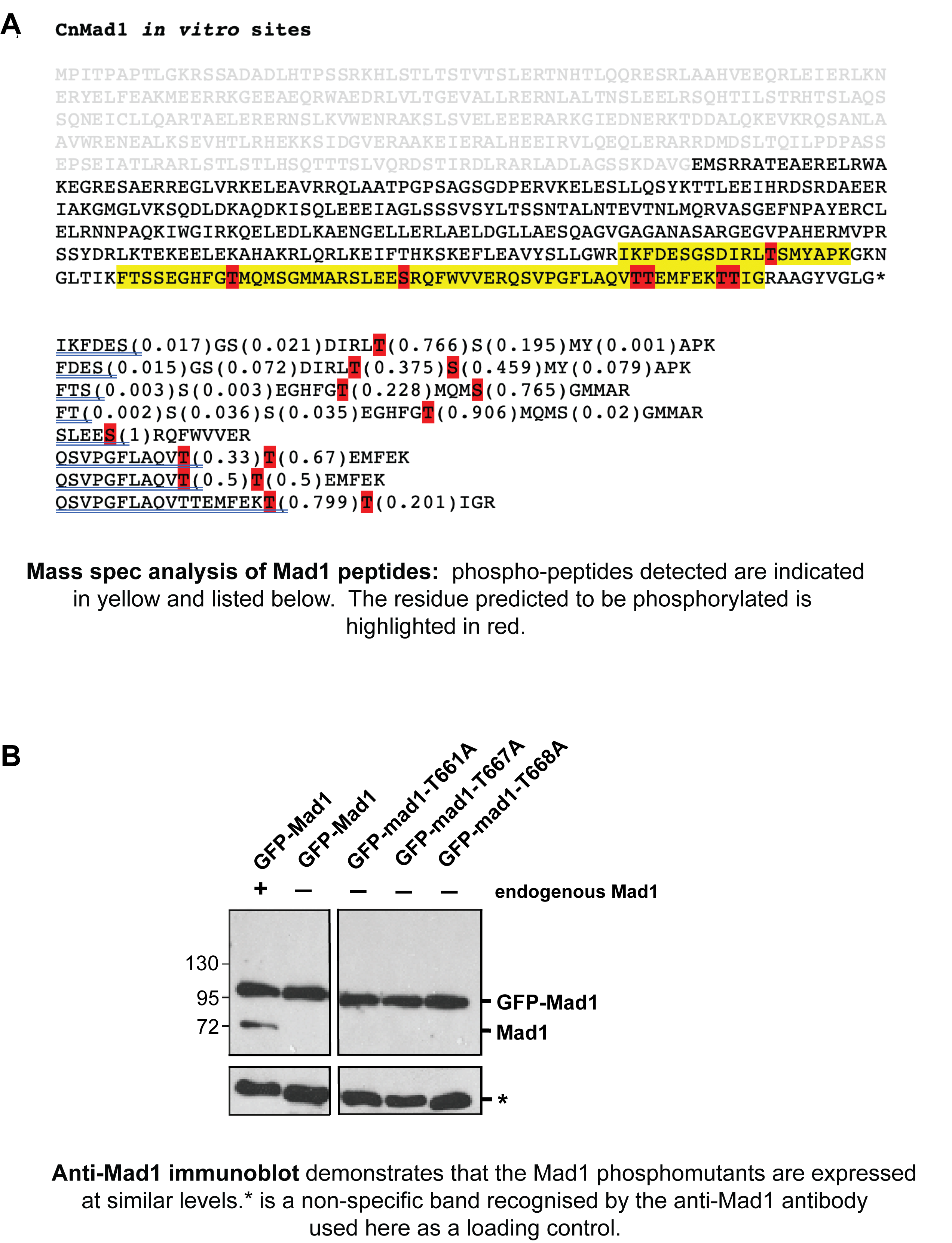

Supplement: S7 Fig — (A) in vitro Mad1 phosphorylation sites detected by mass spectrometry. The C-terminus of Mad1 was phosphorylated in vitro by Mps1 kinase, run on a gel and the Mad1 band excised then digested with trypsin. The phosphopeptides identified are listed and highlighted in yellow on the sequence of Mad1p. The probabilities of specific S/T residue modification are indicated. (B) The mad1 phosphomutant proteins are stable. Immunoblot of whole cell extracts from GFP-mad1 phosphomutants, using the anti-Mad1 antibody. * indicates a cross-reacting band, used here as a loading control. (TIF) [file pgen.1011302.s007.tif]
