# Supplementary material for: Conserved signalling functions for Mps1, Mad1 and Mad2 in the Cryptococcus neoformans spindle checkpoint
Source: PLoS Genet. 2024 Jun 3;20(6):e1011302. doi: 10.1371/journal.pgen.1011302 (PMC11175454; doi:10.1371/journal.pgen.1011302)
Supplement: Attachment — Submitted filename: Aktar_pointbypoint_rebuttal.docx [file pgen.1011302.s008.docx]

We thank all four reviewers for their careful analysis and suggested improvements to our manuscript. We are pleased to submit a revised version here. A point by point rebuttal follows:

Reviewer #1: In the manuscript by Aktar K. et al., the authors characterize the Spindle Assembly Checkpoint (SAC) in Cryptococcus neoformans. The Authors focus on the Mps1 kinase and the downstream checkpoint components Mad1 and Mad2. The Authors provide multiple lines of evidence using complementary experiments that these components act as part of SAC in this species. The Authors conclude that the classic checkpoint functions of the SAC are conserved in Cryptococcus. The Authors also perform purification of GFP-Mad1 followed by mass spectrometric analysis of associated proteins, which reveals not only expected SAC components but also other proteins that can be further investigated. Finally, the Authors quantitated viability of titan cells and their daughters and find that the mad1, mad2 and mps1 mutants exhibit reduced tian cell/titan daughter viability. The Authors propose that C. neoformans Mps1, Mad1 and Mad2 proteins have important, possibly novel, roles in ensuring high fidelity chromosome segregation during stressful conditions.

Comments

1. In the abstract the Authors state that cells lacking Mad1 or Mad2 when treated with microtubule depolymerizing agent “die rapidly”. I suggest the Authors use a more precise way to describe the timing of death, as the time of death is relative.

We have reworded the abstract, to: “and die as a consequence”.
2. The Authors state: “Fig.1D shows that we can rescue the benomyl sensitivity of the mad1Δ strain by complementation with a GFP-Mad1 construct”. I suggest the Authors also add here that this also means that the GFP-Mad1 chimera is likely at least partially functional (that GFP did not abrogate the function of Mad1 when used as a fluorescent tag on Mad1).

We thank the reviewer and have made the suggested change (page 5)
3. In Fig 2D, mad1D on 2.5 concentration of Ben shows some spontaneous mutants that show colony growth – I am curious to see what are those? Any precedence for this from other yeasts?

On occasions we do see ‘suppressors’ appear on such plating experiments. However, in this case I think it was simply ‘splash over’ from the adjacent wells of the 96-well microtitre plates we use to make the serial dilutions.

4. Why did the Authors switched between benomyl and nocodazole in some experiments? It should be explained what is the benefit of using one depolymerizing drug versus the other and not thiabendazole for instance.

This is due to drug solubility and cost issues. Benomyl is cheap and works extremely well in plates (where we add it to boiling agar, due to solubility issues). Thiabendazole could have been used in its place. Nocodazole is expensive and works particularly well in liquid media, where it can be added to the media at room temperature, just before use.
5. In Figure 3B graph, I suggest, the Authors label the Y axis as % large-budded cells and not mention it is an arrest - especially for the mutants it is not known whether those observed large-budded cells are indeed arrested or simply at metaphase-anaphase and continuing the cell cycle.

Thanks. We agree and have made the suggested change.
6. When referring to Fig.S2, the Authors state: “The mad and mps1 mutants all display a rapid rate of death” I suggest the Authors use more precise description instead of “rapid”.

We now state that the mutants die faster than the wild-type control, likely as a consequence of their first division.
7. I suggest for the figure S2 graph, in the label of the X axis, the Authors include "Length of exposure to nocodazole (minutes)”.

Thanks. Agreed, labelling changed.
8. The Authors state: “nuclear pores act as a site of MCC”. I suggest the Authors define the MCC here (Mitotic checkpoint complex).

Agreed – suggested change made.
9. Figure 5 C and D (especially D) need DIC/brightfield images so that individual cells can be identified.

Fig. 5D has been deleted, the experiment repeated and this is now included as S Fig4 (with DIC). Also the Cse4 experiment has been extended to include cycling cells with DIC. Individual cells are outlined in 5C.

10. Is the nuclear periphery localization of Mad1 the same as previously described localization of CENP-A in pre-mitotic C. neoformans cells (does Mad1 localize to centromeres in pre-mitotic/stationary phase cells?)? Even though the Authors test co-localization of Mad1 and Cse4, they don't show non-arrested stationary cells and don't comment on this question.

We thank the reviewer for this interesting question and now include images of cycling cells to address it. From these (new Fig 4E) it is clear that Mad1 does not co-localise with centromeres in interphase cells. The text has been changed accordingly (page 8).

11. The Authors state: “We grew 500mls of yeast in YPD” – space is missing between 500 and mls – mls should be ml? Corrected.
12. The Authors state: “We were surprised to observe that all of these strains (wild-type, mad1 and mad2 died quickly after galactose addition (data not shown).” These two findings are intriguing but not explored in this manuscript. This observation also correlates with and may be related to the low viability of WT cells treated with nocodazole as shown in FigS2.

These points are now addressed in the discussion (page 12).
13. The Authors state: “We conclude that the C-terminal phosphorylation site T667 is an important Mps1 substrate in C. neoformans.” – Up to this point in the manuscript, all the data are robust and well presented but not surprising or novel - C. neoformans SAC seems to function in principle very similar to other already described systems including other (ascomycetous) yeasts. The element of novelty is the fact that this work presents for the first time SAC in basidiomucetous fungus.

We now stress the fact that *C.neoformans* is a model basidiomycete, very evolutionarily distant from the ascomycetes, including *S.cerevisiae* and *S.pombe*. See page 3.

14. The Authors state: “The same experiment reveals that only ~50% of mps1 mutant titan cells were viable and around ~70% of the mad1 and mad2 mutant titans.” I suggest adding … “were viable” at the end of the sentence or moving “were viable” to the end of the sentence. Also, 70% viability of mad1 and mad2 mutants should not be classified as a severe defect. While the defect in mps1 delta cells is more dramatic, defect observed for mad mutants is not severe.

In response to other reviewers, this figure has been removed.
15. The Authors state: “Our interpretation of this experiment is that at least a sub-set of the checkpoint proteins (Mad1, Mad2 and particularly Mps1 kinase) have a novel function that is particularly important for the viability of titan cells and/or their daughters.” This is very intriguing but is not followed upon in this study - it remains unclear what is this presumed function.

In response to other reviewers, this figure has been removed.
16. Regarding temperature sensitivity testing of the mad and mps1 mutants - Perhaps testing in on plates at 39C may show more striking phenotype. Also, it would be interesting to test what happens to those mutant cells upon exposure to 37C/39C? Do they die, or are just arrested?

In response to other reviewers, this figure has been removed.
17. Regarding the experiment and data presented in Fig.9 - As these mutant cells are generally growing poorly at 37C (and potentially may have even more severe growth defect when 5% CO2 and poor nutrient environment is applied), it would be important to ask if upon exposing the cells to titanization protocol also the non-titans are less viable/dead. The Authors micromanipulated and tested specifically the titans but did not test regular cells. It is possible that the observed loss of viability is common to all cells and not specifically titans.

In response to other reviewers, this figure has been removed.
18. The Authors state: “Point mutations that abolish the checkpoint, such as mad1-T667A and bub1-cd1, did not display reduced titan cell viability.” Are those specific two mutants temperature sensitive?

In response to other reviewers, this figure has been removed.
19. The Authors state: “The importance of MAD1, MAD2 and MPS1 in titan cells could be directly related to their increased ploidy.” This is an intriguing idea - however, it should be first tested if non-titans of the SAC defective mutants have reduced viability after the titanization assay (as described in #17) and whether there is significant difference between non-titans and titans in this assay.

We agree and are analysing this in the lab. In response to other reviewers, this figure has been removed.
20. In some parts of text, space is lacking between the number and the unit - for instance in Figure 2C and D panels. Thanks – now corrected.

Reviewer #2: This paper deals with the characterization of some spindle checkpoint proteins (mainly Mad1) in the pathogenic yeast Cryptococcus neoformans. The authors identified MAD1 and MAD2 on the basis of sequence homology and carried out their first functional characterization. As previously shown in other systems, C.n. Mad1 and Mad2 are required for the metaphase arrest in the presence of microtubule-depolymerizing drugs or upon MPS1 overexpression. Additionally, Mad1 localizes at nuclear pores in interphase and relocalizes to kinetochores in mitosis. The study of C.n. Mad1 interactors by mass spectrometry identified several proteins that are known to interact with Mad1 in other systems (e.g. Bub1, Mad2, Cdc20, APC subunits, etc.). Authors identified an RLK motif that is required for Mad1 interaction with Bub1 and for normal benomyl sensitivity (suggesting a likely involvement in the checkpoint). They also identified Mps1-dependent phosphorylation sites in vitro, one which may be important to induce a metaphase arrest upon nocodazole treatment or MPS1 overexpression. Finally, they show that deletion of MAD1, MAD2 or MPS1 significantly impacts the viability of titan cells, which are specialized polyploid cells that C.n. cells produces during human infection and are linked to pathogenicity and drug resistance. This raises the possibility that spindle checkpoint genes may be exploited as potential therapeutic targets.

The paper is well written and the logic easy to follow. Experiments are generally well designed and carefully interpreted. There are however a few points that in my opinion should be addressed to consolidate some observations and strengthen the conclusions.
We thank the reviewer for their positive comments.
Main points:

The data about Mad1 phosphorylation by Mps1 are interesting, but experiments supporting that phosphorylation takes place also in vivo are missing. Since the authors have raised anti-Mad1 antibodies, they could test if Mad1 undergoes a phosphorylation-dependent shift in electrophoretic mobility upon nocodazole treatment, as previously shown by the senior author for budding yeast Mad1. Alternatively, the mass spec data on GFP-Mad1 IPs could be further analyzed to map Mad1 phosphorylation sites and compare them with those obtained in vitro.

We agree that this *in vivo* confirmation (by mass spec) is lacking. However, we believe that the *mad1-T667A* phenotype and similarity of our Cryptococcus data (and the CnMad1 alphafold model) to published human data is consistent with our interpretations.

Unfortunately, further analysis of this mass spec dataset hasn’t proved useful, as the peptide coverage is insufficient for firm conclusions to be drawn. A large scale repeat, along with enrichment of phospho-peptides would likely be needed.

We have looked carefully for a phospho-dependent gel-shift of CnMad1 but unfortunately it is not apparent. Other cryptococcus proteins (eg Spc105 and Mps1) do gel-shift in our experiments, so this is not a simple technical issue. As an aside: we note that our analysis of Mad1 in *S.pombe* also failed to detect gel-shift after phosphorylation.

In Fig. 6A the graph label (cycling vs noc arrested GFP-Mad1) does not match the text on page 8 (tagged vs untagged). If the data refer to the cycling vs noc-arrested condition, which I believe is the case, what is the difference with Fig. 6C? The two graphs look exactly the same.
Also, was the untagged control arrested in nocodazole? The way cells were grown and treated should appear in the figure legend.
Apologies for this confusion. The data is the same, but labelled to focus on different types of interacting proteins. For clarity, we have now moved the nucleoporin interactions to the supplementary data (S Fig5). The untagged control was treated with nocodazole and the figure legends have been improved as requested.

Western blots (Fig. 7C, 7E, 8E) lack a loading control. For instance, in Fig. 7E it seems that MAD1 deletion appreciably reduces the levels of MPS1 overexpression. Is this the case?
The Fig 8E experiment has been repeated and loading controls now included for 7C, 7E. A new loading control for original 8E experiment is now included in S Fig7B.

Minor points:

In Fig. 1D labeling of the first image should be “no benomyl” or “0 ug/ml” instead of “30°C”. Indeed I assume that all plates were incubated at 30°C. Same comment for Fig. 2D, 8D and S3.

Correct – our labelling has now been clarified, as requested.

The style of the two graphs in Fig. 2E should be homogenized. Done.

I assume that Fig. 5B shows the same cell at different stages of mitosis. Wouldn’t it be more logical to invert the order of the images, so that mitosis proceeds from top to bottom, rather than the other way around? We agree, the order has been inverted. Please not that these are not images of a single cell movie, rather representative images from a cycling population.

Why does GFP-Mad1 show a diffuse signal rather than being at nuclear pores in the small budded cell at the bottom row? We have now removed this image as it is not representative of all early mitotic cells. It is possible that GFP-Mad1 would be released from the nuclear envelope, before being recruited to kinetochores.

Were the IPs in Fig. 6F done from nocodazole-treated cells? This information should appear in the figure legend. Yes, all IPs were from nocodazole-treated cells. Apologies for the confusion: the legend now states this clearly.

The Y axis in Fig. 7D is unlabeled. Apologies – now labelled.

In Fig. 8A it is not clear which band corresponds to His-Sumo-Mps1. What is the purpose of having a Sumo tag? The sumo tag aids in solubility of Mad1. This is mentioned in the materials and methods section and the band is labelled clearly now.

Was the graph in Fig. 8B normalized to the amount of Mad1 in each sample (note that Mad1-CT-AA has lower levels than Mad1-CT)? Yes, this was normalised as stated. This is described in the figure legend.

In Fig. 8F the data from the three biological replicates are more dispersed for the T667A mutant than for T667-8A, yet the latter has four asterisks of statistical significance while the latter has only three. Is this correct? Apologies, this was an error and has now been corrected.

Fig. 9C lacks error bars. In response to other reviewers this figure has been deleted.

In Fig. S2 mutants are defined as KO, as opposed to Δ in the text and figure legend. Thanks this is now changed to Δ.

Reviewer #3: In a very nice study, the authors investigate the mechanisms of the spindle assembly checkpoint (SAC) in the fungal pathogen Cryptococcus neoformans. The authors first identify genes in C. neoformans that are orthologous to the SAC effectors MAD1, MAD2, and MPS1. Using microtubule poisons, they go on to demonstrate the requirements for MAD1, MAD2, and MPS1 to arrest cells in response to spindle perturbations. Using co-localization studies and genetic perturbation of specific phosphorylation sites, the authors develop evidence for mechanistic interactions between these effectors and other SAC regulatory proteins and protein complexes. Finally, the authors suggest that in polyploid Titan cells, mad1, mad2, and mps1 mutants have a viability defect.

This is a well-executed and important study revealing a highly conserved mechanism for the SAC in an important fungal pathogen that tops WHO’s list of impactful human fungal pathogens. The results substantially advance our understanding and will be of interest to a broad group of researchers interested in fungal biology and pathogenesis.
We thank the reviewer for their careful review and generous comments.
This group has substantial expertise in uncovering mechanisms in the S. cerevisiae SAC and they have developed creative and relevant approaches to study this mechanism in C. neoformans. That said, there appear to be some implicit assumptions (perhaps made based on work in S. cerevisiae) that are used to reach conclusions that aren’t well supported by the data presented in C. neoformans. In particular, the repeated assertion that Mad1 is recruited to unattached kinetochores in mitotic cells is a very fine-grained conclusion drawn from very coarse-grained co-localization studies. Conclusions should be presented directly from the data, and any implicit assumptions that lead to a specific conclusion should be made explicit. I suspect that the authors are correct, but they need to fill in the gaps in the logic presented that make it difficult for the readers to assess whether the data support the conclusions. More details are presented below.

Finally, there is only one experiment and figure out of 9 total figures related to Titan cells. Thus, the title of the manuscript doesn’t really fit with the majority of the study. I would suggest a change to reflect that the authors are really doing a deep dive on the SAC in C. neoformans. As suggested, we have now deleted this figure.


Specific critiques:

1. The micrographs in Figure 4B are hard to see and interpret. A composite image would be helpful. Agreed – a composite version is now presented, with several cell outlines in Fig 4.

2. In Figure 5B, the authors conclude; “Representative mitotic images demonstrate that GFP- Mad1 is recruited to unattached kinetochores during mitosis (Fig.5B) and enriched there during SAC (nocodazole) arrest (Fig.5C-E). “ I don’t see any evidence in 5B that Mad1 is recruited to unattached kinetochores as there is no marker for kinetochores. What is visible is that Mad1 appears to localize between spindle poles as marked by Tub4-mCherry. We agree: kinetochores will be found between the spindle poles but these images do not prove kinetochore localisation. The images are consistent with this hypothesis. The authors go on to suggest that; “…Mad1 does not co-localise with gamma-tubulin (Fig.5BC). While this may be true in a more fine-grained analysis, there is plenty of colocalization signal (yellow) in the merged images in 5C. We agree. Kinetochores can be closely associated with SPBs and would be expected to overlap with gamma tubulin in this experiment. Our data is consistent with Mad1 localisation to mitotic kinetochores, but does not prove it. Again, the authors should tone down the conclusions and be explicit about any assumptions that might be drawn from “S. cerevisiae” or other systems. We have described our findings in more careful detail, toned down conclusions accordingly, and point out the similarities with other systems (page 9).

3. For Figure 6, the authors do a nice mass spec analysis, but it is not made clear in the text or the figure legend how this was done. The authors state; “To understand more about the molecular interactors of GFP-Mad1 in Cryptococcus neoformans we carried out large scale purifications, from cycling and from checkpoint arrested cells. We grew 500mls of yeast in YPD, added nocodazole for 3 hours, then harvested cells, made whole cell extracts and immunopurified GFP-Mad1 using GFP- TRAP beads.” Apparently they also made a cycling cell extract and an extract from Noc arrested cells that had an untagged Mad1 allele. These are great controls of course, but a little more detailed explanation of the experiment will make it easier for readers to understand. Apologies, this was rather brief – a more careful description of this experiment and the controls is now included in the text. The labelling and figure legends are also improved. That includes an explanation of the significance of being above or below 0 on the x-axis of the volcano plots. I would guess many readers are not familiar with this presentation of the data so an extra sentence of explanation would increase clarity. The significance is explained in more detail now, in the text, methods section and the figure legend.

4. More detail supporting the logic for the conclusions drawn from Fig. 7D would also be helpful. In order to conclude that the arrest due to Mps1 over-expression requires Mad1 and Mad2, it seems lone should compare the % short spindles in Dex media (which should be the baseline measurement of cells not over-expressing Mps1 but have short spindles due to their normal position in the cell cycle) with the mad1 and mad2 deletes in Gal media. That comparison should be shown in the bar graph in 7D so readers don’t have to pull that data from Figure 7B. If one makes that comparison, it seems arguable that the Mad2 contributes to but is not required for some of the O.E. Mps1 arrest.

Thanks, we have now repeated this experiment a number of times, along with the dextrose controls as suggested. New data is plotted in Fig. 7D where we specifically count metaphase cells (rather than large-budded). For this experiment we defined these as being large-budded cells with a single DAPI-stained nucleus in the bud. This is typical for basidiomycetes.

5. The results of a very nice experiment on Titan cells are presented in Figure 9. However, as presented, the impact of deleting SAC genes on the viability on Titan cells is hard to interpret without a couple of controls. First, there is no direct data presented for the effect of these deletions on vegetatively growing haploid cells, so it is not clear if this is a Titan cell specific phenotype. In figure 2E we see that mad1, mad2, and mps1 deletes negatively affect the growth of cells at 30 degrees and even more so at 37 degrees. But we can’t tell from this assay if the deletes are less viable or have a slower division cycle. The spot assay in Figure 2D is not sufficiently quantitative to make a determination about viability. The authors should perform a quantitative viability assay in mutant haploid cells to determine if this viability phenotype is Titan cell specific. Also, given that the SAC deletions have a substantial impact on growth in Fig. 2E, one might argue that stress + SAC mutations lowers viability. What was the impact on viability of growing mutants in stressful conditions during the Titanization protocol? This data might help push these results towards some mechanism.

We thank the reviewer for all these suggestions and agree that these are important experiments. As they suggested, we have now removed these titan studies from the current manuscript. With more time, these will form the basis to a future study, specifically looking at the role(s) of SAC proteins in the polyploid titans.

6. Without some further insight, the data in Figure 9 is just phenomenology and seems like an unrelated addition to the investigation of the SAC mechanism. In my opinion, the data presented in Figure 9 could be removed from the manuscript without affecting the substantial impact of the rest of the study. The authors should consider saving this data as the first figure in a new study addressing the role of the SAC in Titan cell biology. Of course, that would necessitate a change in title, which is likely warranted regardless of the inclusion of Fig. 9. We agree and after discussion with the PLoS editors, have now done this.

Reviewer #4: In the manuscript titled mps1 and mad mutations reduce Cryptococcus neoformans titan cell viability, the authors investigate the well described mitotic checkpoint in this pathogenic yeast species. C. neoformans is a human pathogen that affects a significant global population and therefore understanding mechanisms of weakness for the purpose of therapies is a very important line of questioning. The authors find, not surprisingly, that this yeast exhibits mitotic checkpoint behaviors similar to those in other, more commonplace, yeast models. The paper does an admirable job of testing these, however, again not surprisingly, does not reveal significant new insights into the mitotic checkpoint.
On the whole, I am supportive of important work that has the potential to impact a large global need. However I am somewhat underwhelmed with the results; I wish there were something new to report. Given this, my enthusiasm for publication is only moderate. In any case, the below issues should be addressed prior to the event of publication.
We now stress the fact that *C.neoformans* is a model basidiomycete, very evolutionarily distant from the ascomycetes, including *S.cerevisiae* and *S.pombe*. Whilst not necessarily ‘surprising’ we believe that our studies do significantly extend our knowledge of spindle checkpoint mechanism into the basidiomycetes which, whilst very important to human health and food production (crop pathogens), have received very little attention to date.

1) Figures one and two should be combined. In fact, much of that information should be moved to supplement. Thanks: we have combined Figs 1 and 2 and moved parts to the supplementary data.
2) In figure 4, it is difficult to see the results. Insets with higher magnification (zoomed) images of a single selected nuclei should be included. Thanks for this suggestion - we have done this and outlined cells at lower and higher magnifications.
3) Figure 5, the timelapse in B should be changed such that the top is the first timepoint and the bottom is the last. Also, there should be arrows or some other marking to help the reader interpret. Agreed – this was a mistake and we have flipped the time series. These are representative images taken from a cycling population, not a time-lapse series from a single cell.
