# Supplementary material for: Conserved signalling functions for Mps1, Mad1 and Mad2 in the Cryptococcus neoformans spindle checkpoint
Source: PLoS Genet. 2024 Jun 3;20(6):e1011302. doi: 10.1371/journal.pgen.1011302 (PMC11175454; doi:10.1371/journal.pgen.1011302)
Supplement: Attachment — Submitted filename: Response to reviewers_Aktar.docx [file pgen.1011302.s009.docx]

**Authors response to reviewers’:**

Reviewer #1: The Authors, Aktar K. et al. have addressed all the points/comments I’ve included in my previous review. The revised manuscript is further improved and overall, this is a thorough study that is well put together. The Authors decided to remove the data that relate to the question of the impact of the SAC on formation of titan cells. This leaves the manuscript mostly focused on characterizing the SAC major architecture. The main message in the revised version is that the SAC architecture is conserved in C. neoformans. Below are some additional comments.

1. In the Abstract there are double “that that”.

Thanks, now corrected.

2. A comment to the Authors Summary statement: a pathway that is "extremely well conserved" may not be a good choice for antifungal therapy.

Understood. However, there is significant re-wiring (particularly with respect to Bub1) though that is outwith the context of this paper. We have deleted the word extremely.

3. A considerably large fraction of the Introduction is devoted to Titan cells - as the revised manuscript does not address titan cells, I suggest this fragment is reduced and other aspects are included, for instance the fact that C. neoformans acquires resistance to fluconazole via becoming an aneuploid (which is not necessarily related to formation of titan cells) and SAC may be involved in this process.

Agreed: we have modified the balance of the introduction as suggested, reducing our coverage of titan cells, mentioning seed cells, and expanding our introduction of the relevance of aneuploidy.

4. In the Introduction: I am not sure if emphasizing SAC as a potential drug target is justified here, especially given its apparently high level of conservation.

We have unpublished data that Mps1 kinase inhibitors kill Cryptococcus rather effectively.

Thus, we believe that its description as a potential target is warranted.

5. Fig 1B shows that the GFP-Mad1 is functional while Fig 1C shows that the expression levels of GFP-Mad1 are similar to wt Mad1. To better judge the latter, a loading control would be helpful.

Thanks. We now point out in the figure legend that the cross-reacting band, recognised by the anti-Mad1 antibody (~115kD, labelled *) can be used as a loading control.

6. Why did the complementation of mad2 mutant growth require expression of MAD2 from GAL promoter?

It didn’t, but conditional promoters can be useful. In Fig. 3B we complement the *mad2-* mutant with a variant of this plasmid employing the Mad2 promoter (rather than GAL). This is now referred to on both page 6 and page 7 (the complementation is demonstrated in Fig 1D and Fig 3).

7. In Fig 1, the growth of the double mutant is assessed in panel F, not E.

Thanks – now corrected.

8. A mild ts phenotype of the *mad1* and *mad2* mutants may be more pronounced at 39C

Thank you for this suggestion. We have tested higher temperatures, but as the reference strain (H99) was also struggling the differences weren’t reproducibly enhanced.

9. In Fig S3, it is striking that the WT is much affected by the nocodazole arrest. Was the WT and the mutants also tested with exposure to benomyl instead of nocodazole? The mutants exposed to nocodazole only for 25 min already loose viability dramatically. Is this expected? Or alternatively, there is some additional effect of nocodazole that lowers cell viability. Those comments do not change/question the main conclusion but rather point to an additional phenomenon that may be of interest.

We have repeated this experiment with benomyl and with thiabendazole and find similar impacts. Nocodazole does seem to work particularly well in these liquid experiments, which could be for various reasons including solubility issues.

We agree that the rapid death is interesting and we will be analysing this in more mechanistic detail in future studies. Ideally, we would like to generate a conditional (cold-sensitive) tubulin mutant, for comparison with this rate of death during prolonged mitotic challenges.

Reviewer #2: The authors addressed satisfactorily most of my comments. I just have a couple of minor comments on the text that the authors may want to address before final acceptance.

In Fig. 5E, were the immunoprecipitates split in two different gels to be probed with anti-GFP and anti-Mad1 antibodies? If this is the case it should be specified in the figure legend.
Yes, the IPs were split and then run in parallel – this is now specified in the fig legend.

In Fig. 6E, is the anti-myc Ab really 16B12 or rather 16B11? I think that 16B12 is an anti-HA Ab.
Apologies for this confusion. We actually used 9B11 for panel Fig 6E (from Cell Signalling Technology, #2276) and have corrected this in the figure, and sourced it in materials and methods.

In Fig. S4 it is not clear why Dad2-mCherry is not visible in all cells. A possible explanation could be given in the figure legend. The addition of arrowheads in the images would help the readers to identify early mitotic and anaphase cells.

Thanks. Dad2 (along with other outer KT proteins) is only localised to ‘mature’ mitotic kinetochores in *Cryptococcus* (Ref. 6). This is now explained in the text (page 8) and in the figure legend (S4). Arrows have been added, as suggested.

Fig. S6 seems to have two titles.

Thanks – this is now corrected, and we have expanded the figure legend.

Reviewer #3: The authors provided a very thoughtful and comprehensive response to reviewer comments, and I am quite satisfied with the updates. I have no additional critiques of the manuscript.

Many thanks for these improvements!

Kevin.
